# Identification of miPEP133 as a novel tumor-suppressor microprotein encoded by miR-34a pri-miRNA

**DOI:** 10.1186/s12943-020-01248-9

**Published:** 2020-09-14

**Authors:** Min Kang, Bo Tang, Jixi Li, Ziyan Zhou, Kang Liu, Rensheng Wang, Ziyan Jiang, Fangfang Bi, David Patrick, Dongin Kim, Anirban K. Mitra, Yang Yang-Hartwich

**Affiliations:** 1 The First Affiliated Hospital of Guangxi Medical University, Nanning, 530022 Guangxi China; 2 Department of Obstetrics, Gynecology, and Reproductive Sciences, Yale School of Medicine, New Haven, CT 06510 USA; 3 Department of Radiation Oncology, The First Affiliated Hospital of Guangxi Medical University, Nanning, 530021 Guangxi China; 4 Department of Hepatobiliary Surgery, The First Affiliated Hospital of Guangxi Medical University, Nanning, 530021 Guangxi China; 5 The first affiliated Hospital of Nanjing Medical University, Nanjing, 210029 Jiangsu China; 6 Sheng Jing Hospital of China Medical University, Shenyang, 110004 Liaoning China; 7 Department of Pharmaceutical Sciences College of Pharmacy, University of Oklahoma, Oklahoma City, OK 73117 USA; 8 Indiana University Melvin and Bren Simon Comprehensive Cancer Center, Indianapolis, IN 46202 USA; 9 Indiana University School of Medicine-Bloomington, Bloomington, IN 47405 USA; 10 Yale Cancer Center, New Haven, CT 06510 USA

**Keywords:** miPEP133, Tumor suppressor, miR-34a, Pri-miRNA-encoded protein, Nasopharyngeal carcinoma

## Abstract

**Background:**

Very few proteins encoded by the presumed non-coding RNA transcripts have been identified. Their cellular functions remain largely unknown. This study identifies the tumor-suppressor function of a novel microprotein encoded by the precursor of miR-34a. It consists of 133 amino acid residues, thereby named as miPEP133 (pri-microRNA encoded peptide 133).

**Methods:**

We overexpressed miPEP133 in nasopharyngeal carcinoma (NPC), ovarian cancer and cervical cancer cell lines to determine its effects on cell growth, apoptosis, migration, or invasion. Its impact on tumor growth was evaluated in a xenograft NPC model. Its prognostic value was analyzed using NPC clinical samples. We also conducted western blot, immunoprecipitation, mass spectrometry, confocal microscopy and flow cytometry to determine the underlying mechanisms of miPEP133 function and regulation.

**Results:**

miPEP133 was expressed in normal human colon, stomach, ovary, uterus and pharynx. It was downregulated in cancer cell lines and tumors. miPEP133 overexpression induced apoptosis in cancer cells and inhibited their migration and invasion. miPEP133 inhibited tumor growth in vivo. Low miPEP133 expression was an unfavorable prognostic marker associated with advanced metastatic NPC. Wild-type p53 but not mutant p53 induced miPEP133 expression. miPEP133 enhanced p53 transcriptional activation and miR-34a expression. miPEP133 localized in the mitochondria to interact with mitochondrial heat shock protein 70kD (HSPA9) and prevent HSPA9 from interacting with its binding partners, leading to the decrease of mitochondrial membrane potential and mitochondrial mass.

**Conclusion:**

miPEP133 is a tumor suppressor localized in the mitochondria. It is a potential prognostic marker and therapeutic target for multiple types of cancers.

## Introduction

Non-coding RNAs (ncRNAs) include long non-coding RNAs (lncRNAs, longer than 200 nucleotides) and small non-coding RNAs (sncRNAs, shorter than 200 nucleotides). MicroRNAs (miRNAs) are small ncRNAs consisting of 20–24 nucleotides that negatively regulate the stability and translational efficiency of target mRNAs through binding to the 3′ untranslated regions [1]. Many of them have been demonstrated to play crucial roles in post-transcriptional gene regulation and cancer biology. Genes of miRNAs were assumed to be incapable of encoding proteins. The biogenesis of miRNAs involves the processing of larger primary miRNAs (pri-miRNAs) into shorter pre-miRNAs, and the maturation of pre-miRNA to produce active miRNAs [2]. Recent studies have demonstrated that pri-miRNAs harbor short open reading frames that can encode regulatory peptides, termed miRNA-encoded peptides (miPEPs).

miPEPs that regulate growth and development were first identified in plants. For example, miPEP171b of *Medicago truncatula* and miPEP165a of *Arabidopsis thaliana* are two plant miPEPs that positively regulate the levels of their corresponding miRNAs [3]. The overexpressed miPEPs or synthetic peptides specifically increase the accumulation of the corresponding miRNAs and enhance the inhibition of the miRNA-targeted genes involved in root development. Therefore, it is hypothesized that miPEPs specifically stimulate the transcription of their associated miRNA to induce more pronounced silencing of miRNA-targeted genes. However, it is unclear how miPEPs regulate the transcription machinery. The molecular basis of miPEP specificity and activity are unknown.

In animals, very few miPEPs have been identified till date. Two peptides encoded by lncRNAs were identified as important regulators of muscle physiology [4, 5]. Myoregulin (MLN) is encoded by a skeletal muscle-specific lncRNA. It finely controls calcium uptake by interacting with sarco/endoplasmic reticulum calcium ATPase (SERCA) and plays a critical role in regulating skeletal muscle performance. Another peptide of 34 amino acids named dwarf open reading frame (DWORF) also interacts with SERCA and enhances cardiac muscle contractility. A ncRNA-encoded microprotein named Cancer-Associated Small Integral Membrane Open reading frame 1 (CASIMO1) was recently identified as a 10 kDa protein encoded by a long non-coding RNA (NR_029453). CASIMO1 interacts with squalene epoxidase to regulate lipid droplet clustering and the proliferation of breast cancer cells [6].

miR-34a is a tumor suppressor that inhibits the expression of about 700 target genes [7]. It plays an important role in suppressing tumorigenesis and is downregulated in human cancers [8–10]. The expression of miR-34a is mainly induced by p53 [11–13]. However, accumulating evidence indicates that the level of miR-34a can be regulated in a p53-independent manner. Due to its anti-cancer functions, miR-34a has become one of the first targets of miRNA therapy entering clinical trials [14, 15].

In this study, we report for the first time the identification and functional characterization of a miPEP encoded by the primary transcript of miR-34a. We named it miPEP133 (pri-miR encoded peptide 133) because it consists of 133 amino acid residues. Since miPEP133 is highly expressed in the normal pharynx and significantly downregulated in nasopharyngeal carcinoma (NPC), we utilized the in vitro and in vivo models of NPC to study the role of miPEP133 in tumorigenesis. NPC is a rare type of head and neck cancer with 80,000 incident cases and 50,000 deaths annually [16]. It is more prevalent in southern China, southeastern Asian countries, northern and northeastern Africa, Alaska, and western Canada [17]. NPC is a leading cancer type in Malaysia [18]. NPC is usually sensitive to radiotherapy. If diagnosed at early stage it is considered curable with the 5-year survival rate of about 80% [19]. However, more than 30% of patients will relapse with local recurrence or distant metastases [20]. When NPC progresses into advanced disease, the prognosis is very poor. The clinical management of late stage NPC is extremely challenging. Research on the tumor biology and etiology of NPC is very limited comparing to other more common cancer types. Our findings on the regulation and functions of miPEP133 will advance our knowledge on the roles of miPEPs in tumorigenesis and tumor progression.

## Methods

### Human tissue samples

The NPC study protocol was approved by the Human Research Ethics Committee of the First Affiliated Hospital of Guangxi Medical University. The procedures are in accordance with the Helsinki Decaration of 1975. Written informed consent was obtained from all participants. Normal nasopharyngeal tissues adjacent to the operation areas were collected from 8 patients. Ovarian cancer sample collection was carried out with patient consent and approved by the Human Investigations Committee of Yale University School of Medicine. Tissue samples were immediately frozen in liquid nitrogen after resection and stored at − 80 °C until use. Clinical information regarding the tissue samples is in Additional file 1.

### Cell lines and transfection

Human NPC cell lines HNE3, C666–1, CNE2, CNE1, 5-8F, TWO3, and normal nasopharyngeal cell line NP69 were gifts from Guangxi Medical University Nasopharyngeal Cancer Research Laboratory. SKOV3 and Hela cell lines were obtained from NCI Repository of Tumors and Tumor Cell Lines. The ovarian cancer patient-derived cell lines were provided by Dr. Gil Mor (Yale University). Fallopian tube epithelial cell lines were generated previously [21] and provided by Dr. Ron Drapkin (University of Pennsylvania). Cells were cultured in DMEM containing 10% fetal bovine serum, 100 IU/ml penicillin and 100 mg/ml streptomycin in humidified 5% CO_2_ incubator at 37 °C. Lipofectamine 2000 (Invitrogen, Carlsbad, CA, USA) was used for transfection according to the manufacturer’s instruction. Lentivirus was produced as previously described to establish stable cell lines with miPEP133 overexpression [22]. Puromycin (1 μg/ml, Invitrogen) was used to maintain selective pressure. Details about plasmids used in this study are listed in Additional file 2.

### Antibody production and evaluation

The production of anti-miPEP133 antibody was conducted by GenScript (Piscataway, NJ, USA) as described in Additional file 2. The antibody was evaluated by SDS-PAGE and Coomassie Brilliant Blue staining. The specificity of the produced antibody against miPEP133 was validated by ELISA.

### Western blot and co-immunoprecipitation (co-IP)

Details of western blot and co-IP and the information of antibodies are described in Additional file 2.

### Quantitative PCR (QPCR)

Total RNA was extracted using TRIzol reagent (Invitrogen) and reverse-transcribed using Bulge-LoopTM microRNA specific RTprimers (RiboBio, Guangzhou, China) and M-MLV reverse transcriptase (Promega, Madison, WI, USA). Real-time QPCR was performed on a CFX96TouchTM system (Bio-Rad, Hercules, CA, USA) using SYBR SuperMix (Bio-Rad). Mir-X miRNA qRT-PCR TB Green Kit (Takara Bio, San Francisco, CA) was used to detect miR-34a. RNU6B (U6) or GAPDH were used as internal controls. Relative expression levels were calculated using the 2 − ΔΔCT method. Primers are listed in Additional file 2.

### Mass spectrometry

The gel piece containing the 15 kDa protein band from HEK293 cell lysate was excised and digested into peptide fragments for mass spectrometry analysis. Mass spectrometry was performed as previously described [23]. For the identification of miPEP133-binging proteins, the protein lysate of HEK293 cells expressing flag-labeled miPEP133 or control empty vector was incubated with anti-flag-tag antibody in the co-IP assay. Proteins in the co-IP products were identified by mass spectrometry analysis. The proteins that were identified in the IP product of cells transfected with empty vector were excluded from the list of protein hits as background signals (Additional file 4).

### miPEP133 knockdown with siRNAs and CRISPR/Cas9-mediated miPEP133 deletion

siRNAs were transfected using Lipofectamine RNAiMAX (Invitrogen) following the manufacturer’s instruction. miPEP133 siRNAs were synthesized by Ribo Biotechnology (Guangzhou, China). The sequences of siRNAs and the method of CRISPR/Cas9-mediated miPEP133 deletion are listed in Additional file 2.

### Cell fractionation and RNA/protein extraction

The cytoplasmic and nuclear RNA was extracted from cells using Cytoplasmic and Nuclear RNA Purification kit (Norgen BioTeck, Thorold, ON, Canada). Cell fractionation and protein extraction were performed using Qproteome Mitochondria Isolation kit (Qiagen, Germantown, MD, USA) according to the manufacturer’s instruction.

### Cell proliferation assay

Five thousand cells/well were plated in 96-well plates. Cell viability reagent WST-8 (Abcam, Branford, CT) was added to each well and incubated for 3 h at 37 °C at different time points. Optical density at 450 nm was measured using plate reader.

### Flow cytometry analysis of apoptosis, cell cycle, and mitochondrial membrane potential

Cells were stained according to the instruction of the Annexin V-APC/7-AAD kit (Keygen Biotech, Nanjing, China) and analyzed using FACSLyric Flow cytometry system (BD, Franklin Lakes, NJ) to assess the apoptotic cell populations. Cell cycle status was analyzed by propidium bromide (PI) staining and flow cytometry as detailed in Additional file 2. JC-1 mitochondrial membrane potential assay kit (abcam) was used to stain the cells and assess mitochondrial membrane potential by flow cytometer. A potent mitochondrial uncoupler, carbonyl cyanide chlorophenylhydrazone (CCCP) was used as a positive control.

### Migration assay and invasion assay

For wound healing assay, cells were seeded in 6-well plates, grown to 90% confluence. A scratch wound was made using a pipet tip. Wound width was measured under microscope at 24 h. For the transwell migration assay, cells were seeded into the transwells directly (Corning, Corning, NY, USA) with 0.2 mL serum-free medium. For the invasion assay, cells were seeded into the Matrigel-coated transwells (Corning). The bottom wells were filled with normal growth medium. After 24 h, cells in the upper wells were removed using a cotton swab. The migrating/invading cells at the bottom of the transwells were fixed in 4% polyoxymethylene, stained with crystal violet, and imaged in randomly chosen fields. The stained cells were counted.

### p53 response element luciferase reporter assay

PG13-luc (wt p53 binding sites) was a gift from Bert Vogelstein (Addgene plasmid # 16442; http://n2t.net/addgene:16442; RRID:Addgene_16,442). Renilla luciferase control reporter pRL vector was purchased from Promega (Madison, WI, USA). miPEP-133 or control vector was co-transfected with PG13-luc plasmid. p53 transcriptional activation was evaluated as previously described [24].

### Immunofluorescence (IF) staining and confocal microscopy

Formalin-fixed paraffin-embedded tissue sections were deparaffinized and hydrated. Slides were submersed in 1X citrate unmasking solution and heated in a microwave to incubate at 95–98 °C for 10 min. Cells were cultured on chamber slides, fixed with 4% paraformaldehyde in PBS for 10 min at room temperature, and then washed with PBS. The slides were incubated in blocking buffer (5% normal serum and 0.3% Triton X-100 in PBS) for 60 min at room temperature. Primary antibodies were diluted in antibody dilution buffer (1% BSA and 0.3% Triton X-100 in 1X PBS) and incubated with the slides overnight at 4 °C. Fluorochrome-conjugated secondary antibody was incubated for 1 h at room temperature in the dark. Mount medium with DAPI was used to mount the slides (Cell Signaling, Danvers, MA, USA). The stained slides were imaged using Leica SP8 Laser Scanning Confocal microscope. Terminal deoxynucleotidyl transferase dUTP Nick-End Labeling (TUNEL) staining was performed using In Situ Cell Death Detection kit (Roche, Branford, CT, USA).

### Luminescence-based ATP detection assay

Cells were counted using a hemocytometer. Cell suspension was mixed with the same volume of ATP assay reagent (Promega) to incubate for 10 min. The luminescent signal was measured using GloMax Navigator plate reader (Promega).

### Mouse model

Six-week-old female BALB/c-nude mice (Shanghai Laboratory Animal, Shanghai, China) were subcutaneously injected with 10^7^ C666–1 cells that expressed miPEP133 or control lentivirus vector. Tumor width (W) and length (L) were measured daily. Tumor volume was calculated using formula V = π/6*L*W^2^. Three weeks after injection, mice were euthanized to collect the tumors.

### Statistics

Data were presented as the mean ± standard deviation. All experiments included at least 3 biological repeats. Student’s t-test, two-way ANOVA or chi-square test was used in statistical analysis as specified for each experiment. Kaplan–Meier analysis was employed for the survival analysis of two groups (miPEP133 low and high groups). Survival was defined as time from diagnosis until death or until time last followed. The differences in the survival probabilities were estimated using the log-rank test. *P* values less than 0.05 were considered to be statistically significant.

## Result

### miPEP133 is a microprotein encoded by an ORF in the pri-miRNA of miR-34a

**Fig. 1:**
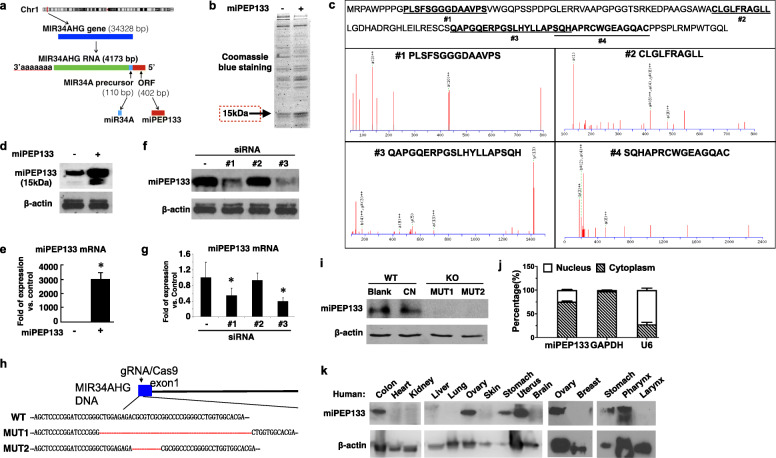
Identification of miPEP133. **a** Schematic graph of the open reading frame (ORF) that encodes miPEP133. The ORF (labeled in red) was identified in the gene of miR34a, *MIR34AHG*. **b** Coomassie blue staining of SDS-PAGE gel that analyzed cell lysate of HEK293 cells transfected with the control plasmid (−) or the plasmid containing miPEP133 ORF (+). **c** Mass spectrometry analysis of the 15KDa protein band excised from SDS-PAGE gel, which confirmed the presence of 4 peptide fragments (#1–4) that match the sequence of miPEP133. **d** Western blot of miPEP133 in HEK293 cells transfected with the control plasmid (−) or the plasmid containing miPEP133 ORF (+). β-actin was used as loading control. **e** The overexpression of miPEP133 in HEK293 cells was assessed at mRNA level by RT-QPCR. Student’s t-test, *p < 0.001. **f** Western blot images of miPEP133 in HEK293 cells transfected with control and 3 siRNAs. siRNAs #1 and #3 knocked down the expression of miPEP133 protein. Student’s t-test, *p < 0.001. **g** RT-QPCR data confirmed the knockdown of miPEP133 mRNA by siRNAs #1 and #3. Student’s t-test, *p < 0.5. **h** Sequencing result demonstrates the CRISPR/Gas9-mediated partial deletion of miPEP133 ORF. Two clones contain the desired deletions of the ORF, MUT1 (28 bp) and MUT2 (5 bp). Red dotted lines indicate the deleted fragments. **i** Western blot images of miPEP133 in HEK293 cells. MUT1 and MUT2 clones both lost the expression of miPEP133 protein comparing to the parent cell line (blank) and the cells transfected with empty vectors (CN). **j** RT-QPCR of miPEP133 mRNA in the nuclear and cytoplasmic fractions of HEK293 cells. U6 and GAPDH were used as control genes. **k** Western blot of miPEP133 protein in normal human tissues

miR-34a is encoded by MIR34AHG gene in the distal region of chromosome 1p. We identified a 402 bp ORF adjacent to the MIR34A precursor sequence region (Fig. 1a). We cloned the ORF into a plasmid vector and transfected HEK293 cells. Using SDS-PAGE and Coomassie blue staining, we detected a band at 15 kDa that was enriched in the ORF-overexpressing cells compared to the control cells (Fig. 1b). We excised the 15 kDa band and digested the proteins into peptides for mass spectrometry analysis. Mass spectrometry analysis confirmed the presence of 4 peptides that matched partial sequences of the ORF protein product (Fig. 1c). This result demonstrates that a 15 kDa protein as the product of this ORF could be stably expressed in cells. We named this protein product as pri-microRNA encodes peptide 133 (miPEP133) since it consists of 133 amino acid residues.

We selected a fragment of miPEP133 (PGPGGTSRKEDPAA) to generate the synthetic peptide-carrier protein conjugate for raising a polyclonal rabbit antibody against miPEP133. Using this antibody in western blot, we detected a 15 kDa band in the cell lysate of HEK293 cells, which intensity was significantly increased in the miPEP133-overexpressing cells (Fig. 1d). We also detected a lower band in the lysate of the miPEP133-overexpressing cells. We postulate that the lower band represents a truncated form of miEPE133 that is cleaved by protease. The overexpression of miPEP133 was also confirmed at RNA level (Fig. 1e).

To verify the specificity of the anti-miPEP133 antibody, we designed three small interfering RNAs (siRNAs) to knock down the endogenous expression of miPEP133 in HEK293 cells. Results of western blot and RT-QPCR showed that two of the siRNAs (#1 and #3) effectively inhibited miPEP133 mRNA expression and significantly decreased the intensity of the 15 kDa protein band that was detected by the anti-miPEP133 antibody (Fig. 1f and g). We also generated two CRISPR/Cas9-mediated deletions of miPEP133, MUT1 and MUT2. They lacked 28 bp and 5 bp in the ORF of miPEP133, respectively (Fig. 1h and Additional file 3: Fig. S1), leading to the loss of the 15 kDa protein band in HEK293 cells (Fig. 1i). These results validated that the 15 kDa protein detected by this antibody was indeed miPEP133. miPEP133 is naturally expressed by HEK293 cells. Cellular fractionation of RNA showed that miPEP133 mRNA mainly localized in the cytoplasm of HEK293 cells (Fig. 1j).

### miPEP133 is expressed in human tissues

Next we assessed the expression of this newly identified protein in different human tissues. Western blot of human tissue homogenates demonstrated that miPEP133 was highly expressed in human colon, ovary, stomach, uterus, and pharynx tissues (Fig. 1k). Similar expression pattern was observed in mouse tissues (Additional file 3: Fig. S2).

**Fig. 2:**
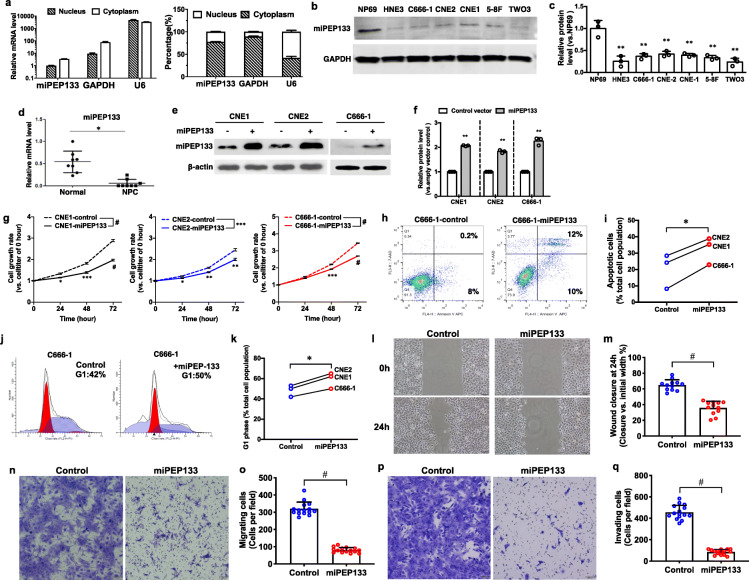
Tumor suppressor functions of miPEP133 in nasopharyngeal carcinoma (NPC) cells. **a** RT-QPCR of miPEP133 mRNA in the nuclear and cytoplasmic fractions of NP69 cells. U6 and GAPDH were used as control genes. **b** Representative images of miPEP133 western blot in NP69 and NPC cell lines. **c** Quantification of miPEP133 band intensity in western blot. Student’s t-test, **p < 0.005. **d** RT-QPCR of miPEP133 in normal pharynx and NPC samples (*n* = 8). Student’s t-test, *p < 0.05. **e** Western blot of miPEP133 in three NPC cell lines transfected with control (−) or miPEP133 plasmid (+). **f** Quantification of miPEP133 band intensity in western blot. Student’s t-test, **p < 0.005. **g** Cell growth rates of NPC cell lines. Two-way ANOVA followed by Sidak’s test, *p < 0.05, **p < 0.005, ***p < 0.001, #*p* < 0.0001. **h** Flow cytometry dot plots of C666–1 cells stained with AnnexinV/7-ADD. **i** Summarized flow cytometry results of AnnexinV/7-ADD-staining in NPC cells lines. Student’s t-test, *p < 0.05. **j** Cell cycle status of C666–1 cells determined by PI staining and flow cytometry. **k** Summarized flow cytometry results of PI-staining in three NPC cells lines. Student’s t-test, *p < 0.05. **l** Representative images of wound healing assay at 0 h and 24 h of control C666–1 cells and the miPEP133-overexpressing C666–1 cells. **m** Quantification of wound closure of control C666–1 cells and the miPEP133-overexpressing C666–1 cells at 24 h. Wound closure was presented as the percentage of wound width that was closed. Student’s t-test, #*p* < 0.0001. **n** Representative images of migrating cells in trans-well assay. **o** Quantification of migrating cells in the transwell migration assay. Student’s t-test, #*p* < 0.0001. **p** Representative images of invading cells in trans-well assay. **q** Quantification of invading cells in the transwell invasion assay. Student’s t-test, #*p* < 0.0001

NP69 is a normal nasopharyngeal epithelial cell line derived from human pharynx tissue. It is non-tumorigenic and exhibits anchorage-dependent growth. NP69 cells expressed miPEP133 mRNA that mainly localized in the cytoplasm (Fig. 2a). Six nasopharyngeal cancer cell lines, HNE3, C666–1, CNE2, CNE1, 5-8F and TWO3 showed significantly reduced levels of miPEP133 protein comparing to NP69 (Fig. 2b and c). In NPC samples we also detected significantly lower levels of miPEP133 mRNA than normal nasopharyngeal tissues (Fig. 2d). These findings led us to hypothesize that miPEP133 was a nasopharyngeal endogenous peptide that was downregulated during malignant transformation, possibly playing a role as a tumor suppressor.

### miPEP133 is a tumor suppressor that inhibits cancer cell proliferation, migration and invasion

To determine whether miPEP133 acts as a tumor suppressor, we overexpressed miPEP133 in three NPC cells lines (Fig. 2e and f). miPEP133 overexpression inhibited the proliferation of NPC cell lines (Fig. 2g), increased the apoptotic cell populations as assessed by AnnexinV and 7-aminoactinomycin D (7-AAD) staining assay (Fig. 2h and i), and induced cell cycle arrest at G1 phase (Fig. 2j and k). In scratch wound healing assays, the migration of C666–1 cells that overexpressed miPEP133 was significantly slower than the control C666–1 cells (Fig. 2l and m). In transwell migration assays, fewer cells migrated in the miPEP133-overexpressing group than the control group (Fig. 2n and o). In transwell invasion assays, the numbers of C666–1 cells that invaded through the Matrigel were significantly reduced in the miPEP133-overexpressing group than the control group (Fig. 2p and q).

### miPEP133 inhibits tumor growth in vivo

**Fig. 3:**
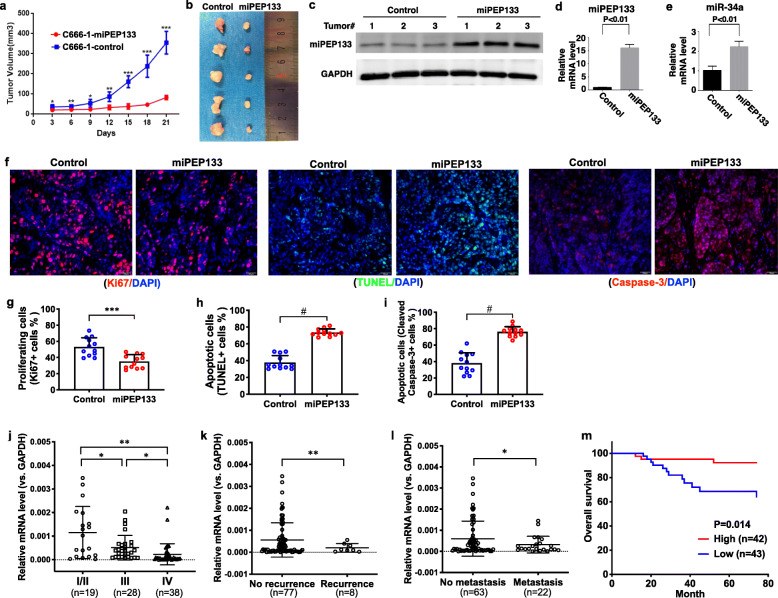
Tumor suppressor functions of miPEP133 in NPC in vivo. **a** Tumor growth curves of nude mice that were injected with control C666–1 cells or miPEP133-overexpressing C666–1 cells. Two-way ANOVA test followed by Sidak test, *p < 0.05, **p < 0.005, ***p < 0.0005. **b** Tumors xenografts from nude mice. **c** Western blot images of miPEP133 in the tumors formed by control C666–1 cells and miPEP133-overexpressing C666–1 cells. **d** QPCR of miPEP133 in tumors. Student’s t-test, *p* < 0.01. **e** QPCR of miR-34a in the tumors. Student’s t-test, *p* < 0.01. **f** Representative images of Ki67, TUNEL, and cleaved caspase-3 staining in tumors from nude mice. DAPI was used to stain the nuclei. **g** Quantification of Ki67 positive proliferating cells in mouse tumors. Student’s t-test, ***p < 0.001. **h** Quantification of TUNEL positive apoptotic cells in mouse tumors. Student’s t-test, #*p* < 0.0001. **i** Quantification of cleaved capsase-3 positive apoptotic cells in mouse tumors. Student’s t-test, #p < 0.0001. **j** Decreased mRNA levels of miPEP133 were associated with advanced NPC in 85 human tumor samples. Student’s t-test, *p < 0.05, **p < 0.005. **k** mRNA levels of miPEP133 were lower in recurrent NPC. Student’s t-test, **p < 0.005. **l** mRNA levels of miPEP133 were lower in metastatic NPC. Student’s t-test, *p < 0.05. **m** High level of miPEP133 mRNA is a favorable prognostic marker for NPC patient. Kaplan–Meier analysis and log-rank test were performed (*p* = 0.014)

We next demonstrated the role of miPEP133 as a tumor suppressor in a xenograft NPC model in vivo. C666–1 cells were subcutaneously injected into the flank of nude mice. The tumor growth of miPEP133-overexpressing C666–1 cells was significantly suppressed when compared to that of the control C666–1 cells (Fig. 3a). Three weeks after the inoculation, tumors collected from the miPEP133-overexpressing group were significantly smaller than those in the control group (Fig. 3b and Additional file 3: Fig. S3). We confirmed the stable overexpression of miPEP133 in the tumor tissues at protein and RNA levels (Fig. 3c and d). Importantly, we identified the upregulation of miR-34a in the tumors overexpressing miPEP133, suggesting that miPEP133 induced the expression of miR-34a (Fig. 3e).

The immunofluorescence (IF) staining of tumor tissues demonstrated that the miPEP133-overexpressing tumors had lower percentages of Ki67-positive proliferating cells, higher percentages of TUNEL positive apoptotic cells, and higher percentages of cleaved-caspase-3-positive apoptotic cells than the control tumors (Fig. 3f, g, h, and i). This result again supports the role of miPEP133 in inhibiting proliferation and inducing apoptosis in cancer cells.

### miPEP133 is a prognostic marker that is downregulated in recurrent and advanced NPC

We evaluated the expression levels of miPEP133 by RT-QPCR and analyzed their relation to clinical characteristics in 85 cases of NPC. No significant association between miPEP expression level and sex, age, smoking history, T staging, N staging, or TNM staging was observed (Additional file 1). The average level of miPEP133 mRNA was higher in stage I/II NPC than that of stage III or stage IV, and stage III NPC expressed higher levels of miPEP133 mRNA than stage IV NPC (Fig. 3j). The downregulation of miPEP133 mRNA was associated with tumor recurrence (Fig. 3k) and metastatic diseases (Fig. 3l). We divided the cases into miPEP133-high and miPEP133-low groups using the mean of miPEP133 mRNA level as a cutoff. The Kaplan-Meier survival analysis and. Log-rank test result suggests that the miPEP133-low group had a significantly lower overall survival rate than the miPEP133-high group (*p* = 0.014, Fig. 3m). These results indicate that the downregulation of miPEP133 is a potential prognostic marker for poor clinical outcomes and advanced diseases.

### miPEP133 interacts with mitochondrial heat shock protein 70 (HSPA9) to regulate mitochondrial function

**Fig. 4:**
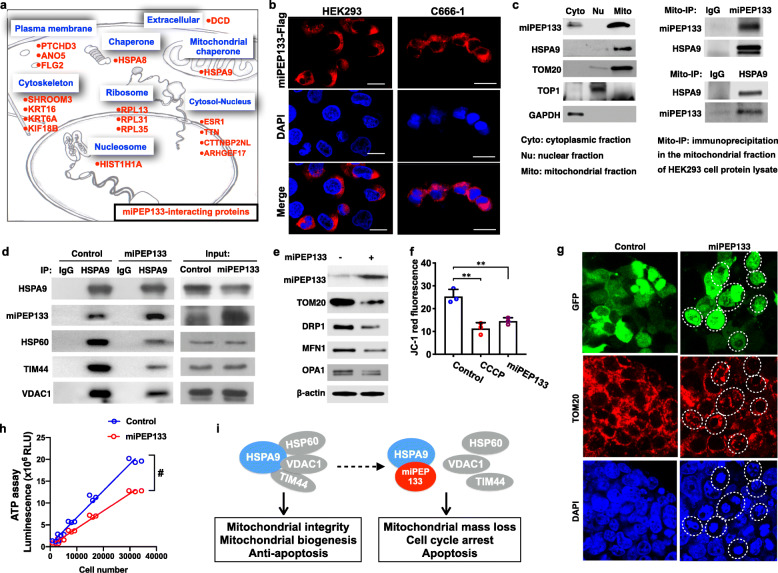
miPEP133 protein function. **a** The miPEP133-interacting proteins in HEK293 cells identified by mass spectrometry. **b** Representative confocal microscopy images of flag-tag-labeled miPEP133 in the cytoplasm of HEK293 and C666–1 cells. **c** Western blot images of miPEP133 in cellular fractions of HEK293 cells and co-IP of miPEP133 and HSPA9 in the mitochondrial fraction. TOM20, DNA topoisomerase 1 (TOP1), and GAPDH were used as loading controls for mitochondrial, nuclear, and cytoplasmic fractions, respectively. **d** Co-IP of HSPA9 and its interacting proteins demonstrated the ability of miEPE133 to block the interaction of HSPA9 to other proteins, including HSP60, TIM44, and VDAC1. **e** Representative images of western blot of mitochondrial proteins in from the control HEK293 cells and the miPEP133-overexpressing HEK293 cells. **f** Summarized flow cytometry result of JC-1-staining in HEK293 cells transfected with control or miPEP133 plasmid. CCCP-treated HEK293 cells were used as positive control. Student’s t-test, **p < 0.005. **g** Representative confocal microscopy images of HEK293 cells transfected with control vector or miPEP133-expressing vector. GFP (green) indicated the cells were transfected with the vectors. TOM20 (red) labeled the mitochondrial outer membrane. DAPI (blue) was used to stain the nuclei. Dotted circles indicate the cells with shrinking nucleus. **h** Cellular ATP level in control and miPEP133-expressing HEK293 cells. Two-way ANOVA, #*p* < 0.0001. **i** Schematic model of miPEP133-regulated mitochondrial integrity and function

In order to better understand the cellular functions of miPEP133, we performed immunoprecipitation (IP) of miPEP133 in HEK293 cells and analyzed the IP product by mass spectrometry. We identified 18 proteins as miPEP133 binding partners, the majority of which are membrane or cytoplasmic proteins (Fig. 4a and Additional file 4). We confirmed the cytoplasmic localization of miPEP133 in HEK293 and C666–1 cells by transfecting HEK293 and C666–1 cells with a plasmid containing miPEP133 fused with flag-tag and detecting the flag-tagged miPEP133 by IF staining (Fig. 4b).

When we analyzed different cellular protein fractions of HEK293 cells, we identified that miPEP133 was predominantly expressed in the mitochondrial fraction and expressed at a much lower level in the cytoplasmic fraction (Fig. 4c). It was noted that one of the partner proteins identified through mass spectrometry analysis, HSPA9 (also named mtHsp70, GRP75, or Mortalin) was also predominantly present in the mitochondrial fraction (Fig. 4c). Using the mitochondrial fraction as input, we performed co-IP of miPEP133 and HSPA9 and confirmed their direct interaction in the mitochondria (Fig. 4c).

HSPA9 modulates the morphology and function of mitochondria [25–27]. We hypothesize that through the interaction with HSPA9 miPEP133 impacts the functions HSPA9 to regulate mitochondrial morphology and function. Indeed, the overexpression of miPEP133 in HEK293 cells significantly decreased the interaction between HSPA9 and its binding partners, HSP60, TIM44 and VDAC1 (Fig. 4d). miPEP133 overexpression also caused the downregulation of mitochondrial proteins, including translocase of outer mitochondrial membrane 20 (TOM20) and the mitochondrial fission/fusion proteins, DRP1, MFN1, and OPA1 (Fig. 4e). The miPEP133-overexpressing cells showed decreased levels of JC-1 staining than the control cells demonstrating a reduction of mitochondrial membrane potential (Fig. 4f). IF staining of TOM20 in the miPEP133-overexpressing cells confirmed the decreased levels of TOM20 expression and revealed more fragmented mitochondria and more shrinking nuclei in the miPEP133-overexpressing cells than the control cells (Fig. 4g). miPEP133-overexpressing cells produced significantly lower levels of ATP than the same numbers of control cells (Fig. 4h). As previously reported, the inhibition of HSPA9 leads to mitochondrial proteolytic stress, mitochondrial fragmentation, reduced mitochondrial mass, and increased apoptosis [26, 28]. Therefore, our findings demonstrate that miPEP133 binds to HSPA9 to prevent HSPA9 from interacting with HSP60, TIM44 and VDAC1, and inhibits the normal function of HSPA9 as a mitochondrial chaperon, which partially accounts for the miPEP133-induced loss of mitochondrial mass, reduction of mitochondrial membrane potential and ATP production, cell cycle arrest and apoptosis (as illustrated in Fig. 4i).

### miPEP133 is transcriptionally regulated by wild-type p53, but not mutant p53

**Fig. 5:**
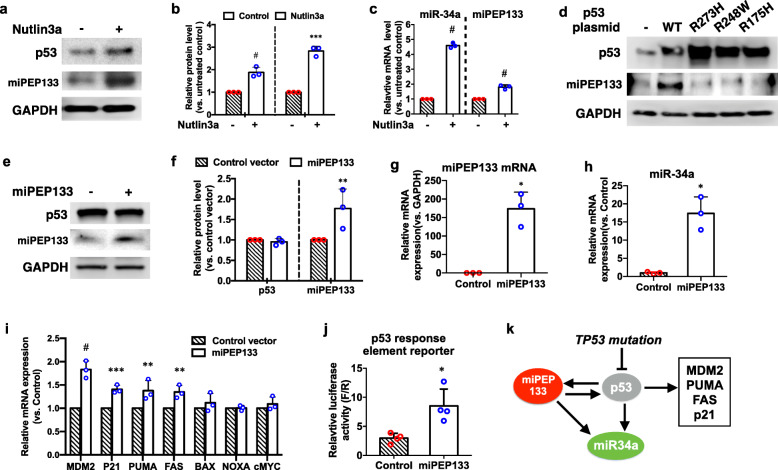
miPEP133 and wild-type p53 reciprocal regulation. **a** Representative western blot images of p53 and miPEP133 in HEK293 cells untreated or treated with Nutlin3a. GAPDH was used as loading control. **b** Quantification of p53 and miPEP133 band intensity in western blot. Student’s t-test, ***p < 0.0005, #*p* < 0.0001. **c** RT-QPCR of miPEP133 and miR-34a mRNA in HEK293 cells untreated or treated with Nutlin3a. Student’s t-test, #*p* < 0.0001. **d** Representative western blot images of wild-type (WT) or mutant p53 and miPEP133 in HEK293 cells transfected with WT or mutant p53 plasmids. GAPDH was used as loading control. **e** Representative western blot images of p53 and miPEP133 in control and miPEP133-overexpressing HEK293 cells. GAPDH was used as loading control. **f** Quantification of p53 and miPEP133 band intensity in western blot. Student’s t-test, **p < 0.005. **g** RT-QPCR of miPEP133 mRNA in control and miPEP133-overexpressing HEK293 cells. Student’s t-test, *p < 0.05. **h** RT-QPCR of miR-34a in control and miPEP133-overexpressing HEK293 cells. Student’s t-test, *p < 0.05. **i** RT-QPCR of p53 target genes in control and miPEP133-overexpressing HEK293 cells. Two-way ANOVA followed by Sidak’s test, **p < 0.005, ***p < 0.001, #*p* < 0.0001. **j** p53 response element luciferase reporter assay in control and miPEP133-overexpressing HEK293 cells. Student’s t-test, *p < 0.05. **k** Schematic model of the reciprocal regulation between miPEP133 and p53

The expression of miR-34a is regulated by p53 through the regions proximal to MIR34AHG gene promoter that contain multiple canonical p53 binding motifs [11, 12]. Since the ORF of miPEP133 is adjacent to miR-34 precursor as part of miR-34a pri-miRNAs (Fig. 1a), we hypothesized that the expression of miPEP133 was also regulated by p53 through MIR34AHG promoter. To test this hypothesis, we first activated p53 with a small-molecule MDM2 antagonist, nutlin-3a, to assess its impact on miPEP133 expression. Our results showed that nutlin-3a treatment for 24 h increased the protein levels of p53 and miPEP133 in HEK293 cells (Fig. 5a and 5b). In addition, miR-34a and miPEP133 mRNA were both upregulated by nutlin-3a treatment (Fig. 5c). Second, we overexpressed wild-type p53 and three common hot-spot mutations of p53 (R175H, R248W, and R273H) in HEK293 cells. The overexpressed wild-type p53 induced the upregulation of miPEP133 protein, but the mutant p53 did not affect the levels of miPEP133 (Fig. 5d). These results suggest that wild-type p53 activates miPEP133 transcription. Mutations can impair the ability of p53 to regulate miPEP133 transcription.

### miPEP133 promotes p53 transcriptional activation

**Fig. 6:**
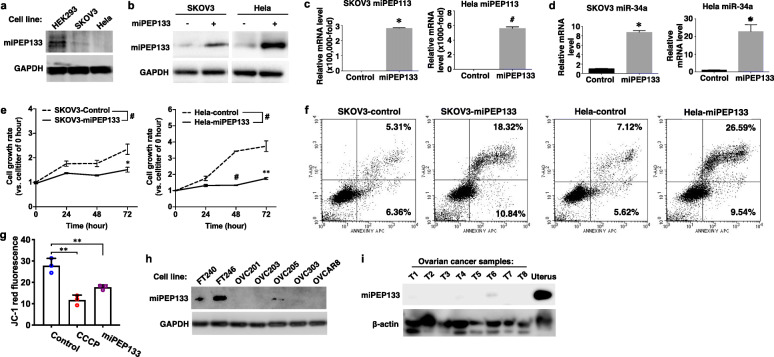
miPEP133 has p53-independent functions. **a** Representative western blot images of miPEP133 in ovarian cancer cell line SKOV3 and cervical cancer cell line Hela. GAPDH was used as loading control. **b** Representative western blot images of miPEP133 in control and miPEP133-overexpressing SKOV3 and Hela cells. **c** RT-QPCR of miPEP133 in control and miPEP133-overexpressing SKOV3 and Hela cells. Student’s t-test, *p < 0.01, #*p* < 0.0001. **d** RT-QPCR of miR-34a in control and miPEP133-overexpressing SKOV3 and Hela cells. Student’s t-test, *p < 0.01. **e** Cell growth rates of SKOV3 and Hela cells. Two-way ANOVA followed by Sidak’s test, *p < 0.05, **p < 0.005, ***p < 0.001, #*p* < 0.0001. **f** Flow cytometry dot plots of SKOV3 and Hela cells transfected with control or miPEPqee plasmid and stained with AnnexinV/7-ADD. **g** Summarized flow cytometry result of JC-1-staining in Hela cells transfected with control or miPEP133 plasmid. CCCP-treated Hela cells were used as positive control. Student’s t-test, **p < 0.005. **h** Representative western blot images of miPEP133 in patient-derived ovarian cancer cell lines, OVC201, OVC203, OVC205 and OVC303. Normal human fallopian tube epithelial cell lines, FT240 and FT246, were used as noncancerous control cells. GAPDH was used as loading control. **i** Representative western blot images of miPEP133 in ovarian cancer samples (T1-T8. Human normal uterus tissue protein lysate was used as a positive control in western blot. β-actin was used as loading control

The overexpression of miPEP133 in HEK293 cells did not change the level of p53 protein (Fig. 5e, f, and g). However, the overexpression of miPEP133 increased the transcription of miR-34a (Fig. 5h) and the other p53 target genes, including MDM2, P21, PUMA, and FAS, which are involved in cell cycle arrest and apoptosis (Fig. 5i). In a p53 response element reporter assay, we identified that miPEP133 overexpression enhanced the p53 transcriptional activity (Fig. 5j). Taken together (as illustrated in Fig. 6k), miPEP133 is transcriptionally activated by wild-type p53. Mutations can impair the ability of p53 to induce miPEP133 transcription. miPEP133 in turn enhances p53 transcriptional activity without changing the level of p53 protein. miPEP133 induces the transcription of its associated miRNA, miR-34a.

### miPEP133 possesses p53-independent tumor suppressor functions

In order to examine the function of miPEP133 in different cellular contexts, we determined the effect of miPEP133 overexpression in an ovarian cancer cell line SKOV3 that is p53 null and Hela cell line that is known for having p53 inactivated by human papillomavirus proteins. In SKOV3 and Hela cells, the levels of miPEP133 protein were significantly lower than that of HEK293 cells (Fig. 6a). We overexpressed miPEP133 in SKOV3 and Hela cells as confirmed at protein and RNA levels (Fig. 6b and c). The overexpression of miPEP133 induced the upregulation of miR-34a in SKOV3 and Hela cells (Fig. 6d), which is consistent with the observation in HEK293 and NPC cells. The overexpression of miPEP133 also significantly decreased the cell viability of SKOV3 and Hela cells (Fig. 6e) and increased the AnnexinV positive apoptotic cell population (Fig. 6f). The JC-1 staining signals were lower in Hela cells that overexpressed miPEP133 that the control cells indicating the reduction of mitochondrial membrane potential (Fig. 6g). These results demonstrated that in the absence of functional wild-type p53 miPEP133 still could act as a tumor suppressor to suppress cancer proliferation, induce apoptosis, and decrease mitochondrial membrane potential. The levels of miPEP133 protein in ovarian cancer line OVCAR8 and four patient-derived epithelial ovarian cancer cell lines (OVC201, OVC203, OVC205 and OVC303) were very low or undetectable, in contrast, two immortalized normal human fallopian tube epithelial cell lines (FT240 and FT246) expressed high levels of miPEP133 protein (Fig. 6h). The fallopian tube epithelial cells have been identified as one of the cell-of-origins for high-grade serous ovarian carcinoma that is the most common type of ovarian cancer. We also confirmed the loss of miPEP133 in eight tumor tissue samples from epithelial ovarian cancer patients (Fig. 6i). These results suggest that miPEP133 is also a tumor suppressor in ovarian cancer.

## Discussion

In this study, we have identified a novel microprotein that is encoded by an ORF in the precursor of miR-34a. This microprotein, namely miPEP133, mainly localizes in the mitochondria and plays a role as a tumor suppressor with the activity to induce cell cycle arrest, cell growth inhibition, and apoptosis in cancer cells in addition to slowing down cancer cell migration and invasion. We revealed the mechanisms of its function that involves interacting with mitochondrial chaperon HSP9A and disrupting the interaction of HSP9A with other proteins. Our findings demonstrated the tumor suppressor activity of miPEP133 and its potential value as a prognostic marker and therapeutic target.

miPEPs are more common in plant, but rare in animals. miPEP133 is the first miPEP that is identified in the pri-miRNA of tumor suppressor miRNA in human. It shares the same promoter as its associated miRNA, miR-34a. Therefore, the expression patterns of miPEP133 are similar to miR-34a. miPEP133 and miR-34a are both downregulated in tumor tissues and both regulated by p53. The other inducers of miR-34a, such as ELK1 and TAp73 [29], may also induce miPEP133 expression, which is yet to be determined in the future studies. The target genes of miR-34a are involved in oncogenic signaling pathways, such as cell proliferation (e.g. cyclins, cyclin-dependent kinases, MYCN, NOTCH1, and MDMX), anti-apoptosis (e.g. BCL2 and SIRT1), cancer stem-like cell properties (e.g. CD44, NANOG, and SOX2), metastasis (e.g. SNAI1 and MET), and immune evasion (e.g. PD-L1). miPEP133 can promote the transcription of miR-34a to enhance the silencing of these miR-34a-targeted genes. We have observed the miPEP133-induced downregulation of ATF3, AXIN2, CDKN1A, DLL1, E2F1, E2F2, FOXP1, JAG1, MYCN, SIRT1, and SOX2, which are miR-34a-targeted genes (data not shown).

The mechanisms underlying miPEP133-induced expression of miR-34a may be explained by different co-existing mechanisms under different cellular contexts. In cells with wild-type functional p53, such as HEK293 cells, miPEP133 can disrupt mitochondrial functions, which activates wild-type p53 transcriptional activity consequently inducing miR34a expression. In the meantime, the miPEP133-induced mitochondrial dysfunction can activate other transcriptional regulators of miR-34a, such as TAp73, to directly bind to the promoter of miR34a [29, 30]. The latter mechanism may be responsible for the miPEP133-induced upregulation of miR-34a in p53-null cancer cells like ovarian cancer cell line SKOV3 [31] and cancer cells without functional wild-type p53. p53 mutation is very common in ovarian cancer, particularly in high-grade serous ovarian carcinoma. NPC cells usually have wild-type p53, however, NPC is highly associated with Epstein-Bar virus that can inactivate p53 [32]. Cervical cancer Hela cell line is also known for having p53 inactivated by human papillomavirus proteins [33]. Our findings demonstrated the p53-dependent and p53-independent tumor suppressor roles of miPEP133 in different cellular contexts with diverse p53 status.

HSPA9 is involved in stress response, antigen processing, control of cell proliferation, differentiation and tumorigenesis [34]. HSPA9 has anti-apoptotic and pro-proliferative activities [35, 36]. Increasing levels of HSPA9 permit cells to survive lethal conditions [37–39]. HSPA9 influences the function, dynamics, morphology, and homeostasis of mitochondria as the only ATPase component of the mitochondrial protein import machinery [35, 40, 41]. Mitochondrial protein precursors are chaperoned by HSPA9 into the mitochondrial matrix with the assistance of co-chaperones [42]. HSPA9 forms a complex with HSP60 and floats freely in the mitochondrial matrix to regulate protein folding [43]. HSPA9 also binds the translocase of the mitochondrial inner and outer membranes [44, 45]. Their complexes control the translocation of precursor proteins and their distribution in the matrix and across the mitochondrial membranes [46–48]. HSPA9-VDAC1 complex modulates voltage-dependent anion-selective channel properties [49]. The interaction with miPEP133 prevents HSPA9 from acting as a chaperon and interacting with its partner proteins, which demonstrates a new mechanism for regulating mitochondrial morphology and function.

## Conclusion

We have identified a novel microprotein encoded by the precursor of miR-34a, miPEP133. It is a tumor suppressor that induces apoptosis in cancer cells and inhibits their migration and invasion. Low miPEP133 expression is a prognostic marker of advanced and metastatic NPC. We demonstrated the reciprocal regulation between wild-type p53 and miPEP133. miPEP133 localizes in the mitochondria to prevent HSPA9 from interacting with its binding partners and regulate mitochondrial function. A better understanding of miPEP133 function and regulation will provide a new biomarker and potential therapeutic target for improving the prognosis and treatment of NPC and other cancers. Our work also sets a foundation for understanding the roles of miPEPs in the tumorigenesis of all cancer types.

## Supplementary information

**Additional file 1.** Basic clinicopathological characteristics of nasopharyngeal carcinoma patients and ovarian cancer tumor sample information.

**Additional file 2.** This section describes the details of experimental procedures and materials that are not described in the Method Section, including Supplemental methods and lists of primers, antibodies, and plasmids.

**Additional file 3.** Description of data: This file includes Supplemental Figures S1-S5

**Additional file 4.** This Excel file contains 3 sheets. They are the lists of proteins identified in control cells, proteins identified in miPEP133-overexpressing cells, and miPEP133-binding proteins confidently identified by excluding the backgrounds in the control cells.

## Data Availability

The datasets supporting the conclusions of this article are included within the article and its additional files. The following is a list of the additional files.

## Abbreviations

miPEP133: Pri-microRNA encoded peptide 133
NPC: Nasopharyngeal carcinoma
HSPA9: Heat Shock Protein Family A Member 9, also known as mitochondrial heat shock protein 70kD
ncRNAs: Non-coding RNAs
lncRNAs: Long non-coding
sncRNAs: Small non-coding RNAs
miRNAs: MicroRNAs
miPEPs: MiRNA-encoded peptides
MLN: Myoregulin
SERCA: Sarco/endoplasmic reticulum calcium ATPase
DWORF: Dwarf open reading frame
CASIMO1: Cancer-Associated Small Integral Membrane Open reading frame 1
co-IP: Co-immunoprecipitation
QPCR: Quantitative PCR
CCCP: Carbonyl cyanide chlorophenylhydrazone
TUNEL: Terminal deoxynucleotidyl transferase dUTP Nick-End Labeling
BSA: Bovine serum albumin
PBS: Phosphate-buffered saline
IF: Immunofluorescence
ATP: Adenosine triphosphate
siRNAs: Small interfering RNAs
CRISPR: Clustered regularly interspaced short palindromic repeats
Cas9: CRISPR-associated protein 9
IP: Immunoprecipitation
HSP60: Heat shock protein 60
TIM44: Translocase Of Inner Mitochondrial Membrane 44
VDAC1: Voltage Dependent Anion Channel 1
TOM20: Translocase of Outer Mitochondrial membrance 20
DRP1: Dynamin-Related Protein 1
MFN1: Mitofusin 1
OPA1: OPA1 Mitochondrial Dynamin Like GTPase
JC-1: 5,5,6,6-Tetrachloro-1,1,3,3-tetraethylbenzimidazolylcarbocyanine iodide
MDM2: Mouse double minute 2 homolog
P21: Cyclin Dependent Kinase Inhibitor 1A, encoded by the CDKN1A gene, also known as Cip1
PUMA: p53 upregulated modulator of apoptosis, also known as Bcl-2-binding component 3
FAS: Fas Cell Surface Death Receptor
ELK1: ETS Transcription Factor ELK1
TAp73: Tumor protein p73
NOTCH1: Notch Receptor 1
MDMX: also known as MDM4, Mouse Double Minute 4, Human Homolog Of; P53-Binding Protein
BCL2: BCL2 Apoptosis Regulator
SIRT1: Sirtuin 1
NANOG: Nanog Homeobox
SOX2: SRY-Box Transcription Factor 2
SNAI1: Snail Family Transcriptional Repressor 1
MET: MET Proto-Oncogene, Receptor Tyrosine Kinase
PD-L1: Programmed Cell Death 1 Ligand 1, also known as CD274 Molecule
ATF3: Activating Transcription Factor 3
AXIN2: Axin 2
CDKN1A: Cyclin Dependent Kinase Inhibitor 1A
DLL1: Delta Like Canonical Notch Ligand 1
E2F1: E2F Transcription Factor 1
E2F2: E2F Transcription Factor 2
FOXP1: Forkhead Box P1
JAG1: Jagged Canonical Notch Ligand 1
MYCN: MYCN Proto-Oncogene, BHLH Transcription Factor
