# Supplementary material for: Identification of miPEP133 as a novel tumor-suppressor microprotein encoded by miR-34a pri-miRNA
Source: Mol Cancer. 2020 Sep 14;19:143. doi: 10.1186/s12943-020-01248-9 (PMC7489042; doi:10.1186/s12943-020-01248-9)
Supplement: Supplementary file 1 — Additional file 1. Basic clinicopathological characteristics of nasopharyngeal carcinoma patients and ovarian cancer tumor sample information. [file 12943_2020_1248_MOESM1_ESM.docx]

**Additional file 1. Table of clinical sample information**

(Note: Tumor and non-tumor samples used in this study were confirmed by pathological examinations. Pathological stage, grade, and nodal status were determined by an experienced pathologist according to the 2002 AJCC/UICC staging classifications.)

**Table 1. Basic clinicopathological characteristics of nasopharyngeal carcinoma patients**

|  |  |  |
| --- | --- | --- |
| Clinicopathologic factors | Overall(n=85) |  |
| Gender |  |  |
| Male | 61 |  |
| Female | 24 |  |
| Age at dignosis |  |  |
| ≤40 | 31 |  |
| ＞40 | 54 |  |
| T staging |  |  |
| T1 | 6 |  |
| T2 | 21 |  |
| T3 | 37 |  |
| T4 | 21 |  |
| N staging |  |  |
| N0 | 10 |  |
| N1 | 34 |  |
| N2 | 33 |  |
| N3 | 8 |  |
| TNM staging |  |  |
| I+II | 19 |  |
| III | 28 |  |
| IV | 38 |  |
| Recurrence |  |  |
| Yes | 8 |  |
| No | 77 |  |
| Distant metastases |  |  |
| Yes | 22 |  |
| No | 63 |  |
|  |  |  |

**Table 2. Ovarian cancer tissue samples.**

| **Patient**  **Number** | **Histology** | **Stage** | ***TP53* status** |
| --- | --- | --- | --- |
| 1 | Clear cell ovarian carcinoma | IVb | S240T |
| 2 | Poorly differentiated ovarian serous papillary carcinoma | IIIc | Wild type |
| 3 | Undifferentiated ovarian serous carcinoma | IIIc | S241F |
| 4 | Ovarian serous papillary adenocarcinoma | IIIc | Wild type |
| 5 | Moderately differentiated ovarian serous adenocarcinoma | IIIc | Wild type |
| 6 | Peritoneal serous papillary carcinoma | IVa | R283C |
| 7 | Differentiated fallopian tube serous carcinoma | IIIc | Wild type |
| 8 | Ovarian serous papillary carcinoma | IIIc | Wild type |
