# Supplementary material for: Identification of miPEP133 as a novel tumor-suppressor microprotein encoded by miR-34a pri-miRNA
Source: Mol Cancer. 2020 Sep 14;19:143. doi: 10.1186/s12943-020-01248-9 (PMC7489042; doi:10.1186/s12943-020-01248-9)
Supplement: Supplementary file 2 — Additional file 2. This section describes the details of experimental procedures and materials that are not described in the Method Section, including Supplemental methods and lists of primers, antibodies, and plasmids. [file 12943_2020_1248_MOESM2_ESM.docx]

**Additional file 2. Supplemental methods.**

*Summary: This section includes the details of experimental procedures and materials that are not described in the Method Section*

**1) Supplemental methods:**

**Anti-miPEP133 antibody production**

For immunization the miPEP133 synthetic peptide was used. Keyhole Limpet Hemocyanin (KLH, ThermoFisher Scientific) was used as a carrier protein to guarantee the immunogenicity of the peptide, which was linked to it using m-maleimidobenzoyl-N-hydroxysuccinimide ester (MBS, Sigma-Aldrich). Freund’s Complete Adjuvant (FCA, Sigma-Aldrich) was used to increase the immune response in the animals. KLH-PEP complex was formed following the protocol for coupling with heterobifunctional reagent according to Coligan. Before immunization, KLH-PEP solution was mixed with an equal volumen of FCA to make an emulsion. The emulsion was injected subcutaneosly in the back of each rabbit, in 4 different sites, on days 0. Animals were also boosted with KLH-PEP solution on days 14 (boost 1), 35 (boost 2) and TBD (final boost). Serum was obtained on days -4 (pre-immune serum, PIS), 21, 42 and 7 days after final boost (immune serum, IS). The final doses used in each immunization was 200 μg KLH-PEP/animal.

In all cases, blood samples were taken from marginal ear vein after animals were anesthetized with ketamin/xylacin. Immune serum was obtained and immunoglobulin G (IgG) fraction was purified by precipitation using saturated ammonium sulfate solution (SAS). The serum was filtrated (nylon filter 0.45 µm) and SAS was added gradually up to a 40% concentration, precipitation was overnight at 4 °C with continuous stirring. The suspension was centrifuged at 10,000g for 30 min and the pellet was resuspended in phosphate buffer saline (PBS) and dialysed in PBS for 4 h at 4 °C in order to remove the (NH4)2SO4. The production and purity of IgG was evaluated by sodium dodecyl sulfate polyacrylamide gel electrophoresis (SDS-PAGE, MiniProtean, BioRad). We used acrylamide 12% resolving gel and 4% stacking gel. After 1-hour electrophoresis the gel was stained with Coomassie Brilliant Blue. The specificity of the produced antibody against miPEP133 was validated by ELISA.

**Western blot**

Total proteins were extracted from cells or tissues by RIPA reagent, protein concentration was calculated, sample was mixed with 5× loading buffer, at final concentration of 1×. Denature all samples at 100℃ for 10 minutes. Samples were subjected to sodium dodecyl sulfate-polyacrylamide gel electrophoresis (SDS-PAGE) separation, followed by transfering onto a PVDF membrane (Millipore, Billerica, MA, USA). Then blocked with 5% nonfat dry milk or 3% BSA for 2 h at room temperature and then incubated with corresponding antibodies follow the instructions, overnight at 4℃, followed by incubation with peroxidase-conjugated secondary antibody for 1h at room temperature. Final signal were detected with as enhanced chemiluminescence detection reagent (Pierce, Minneapolis, MN, USA). Antibodies used in this study were listed in the antibody list below.

**CRSIPR/Cas9-mediated miPEP133 knockout**

To establish miPEP133 knockout stable cell line, we designed single guided RNA (sgRNA) sequences using CRISPR design tool [crispr.mit.edu](http://crispr.mit.edu/). sgRNA sequences are available upon request. Annealed double stranded sgRNA oligos were ligated to the lentiCRISPR vector 2 (Plasmid #52961 http://www.addgene.org/52961/ deposited by Dr. Feng Zhang, Camridge, MA) at a ribonucleoprotein complex which expressed both Cas9 and sgRNA. To produce infectious transgenic lentivirus, the transfer plasmid was transfected into HEK293 cells together with packaging plasmid and envelope plasmid. Supernatant containing virus was concentratd by super-centrifugation. Cells were infected with lentivirus and selected by puromycin (1 μg/ml) for two weeks. The knockout efficiency was confirmed by western blot.

**Co-immunoprecipitation (co-IP)**

SureBeads (Bio-rad) were washed with PBS-T buffer (PBS + 0.1% Tween 20) three times and incubated with an antibody (1 µg) for 10 min at room temperature. Beads were washed with PBS-T buffer three times before the antibody-conjugated beads were ready for IP. Cell lysate was added to the beads and rotated for 1 hour at room temperature. Beads were magnetized to discard supernatant and thoroughly washed with PBS-T for 5 times. Laemmli buffer (40 µl) was added to the beads and incubated for 5 minutes at 90°C. Finally, beads were magnetized to move eluent to a new tube. The eluent was used for western blot analysis.

**Cell cycle analysis**

Cells were collected 1×10^6^ cells 48 h after transfection. The cells were collected by centrifugation, the supernatant was discarded, the cells were washed twice with cold PBS, precooled with 70% ethanol, fixed overnight at 4 ° C, or fixed at -20 ° C for a long period of time. The cells were collected by centrifugation, washed once with 1 mL of PBS, and added with 500 uL PBS containing 50 μg/mL of propidium bromide (PI), 100 μg/mL RNase A, 0.2% Triton X-100 and incubated in the dark at 4 ° C for 30 minutes. Flow cytometry was performed using FACSLyric Flow cytometry system (BD, Franklin Lakes, NJ). Results are analyzed using the ModFit cell cycle software.

**2) List of primers:**

miPEP133-forward: 5′-CTCGGTGACCACGCAGATC-3′;

miPEP133-reverse: 5′-GCAGGTAGTGCAGGCTTCC-3′;

miR-34a: 5’-TGGCAGTGTCTTAGCTGGTGT-3’

(mRQ3’ Primer for miR-34a was supplied in the Mir-X miRNA qRT-PCR TB Green Kit)

GAPDH-forward: 5′-GCATGGGTCAGAAGGATTCCT-3′;

GAPDH-reverse: 5′-TCGTCCCAGTTGGTGACGAT-3′;

MDM2-forward: 5′-TGGTGAGGAGCAGGCAAATGTGC-3′;

MDM2-reverse: 5′-ACCAGGGTCTCTTGTTCCGAAGC-3′;

P21-forward: 5′-AACCGGCTGGGGATGTCCGT-3′;

P21-reverse: 5′-CTGCTCGCTGTCCACTGGGC-3′;

PUMA-forward: 5′- GCGGACGACCTCAACGCACA -3’;

PUMA -reverse: 5′-GGCTCTGTGGCCCCTGGGTA-3′;

FAS-forward: 5′- CAGACTGCGTGCCCTGCCAA-3’;

FAS -reverse: 5′-TCTGGGTCCGGGTGCAGTTT-3′;

BAX-forward: 5′-TGGCAGCTGACATGTTTTCTGAC-3’

BAX-reverse: 5′- TCACCCAACCACCCTGGTCTT-3′;

NOXA-forward: 5′- GCGCGCAAGAACGCTCAACC-3′;

NOXA -reverse: 5′- TGCCGGAAGTTCAGTTTGTCTCCA-3′;

cMYC-forward: 5′- ACTCTGAGGAGGAACAAGAA-3′;

cMYC -reverse: 5′- TGGAGACGTGGCACCTCTT-3′.

**3) List of antibodies:**

| **Target** | **Host species/**  **conjugation** | **Application** | **Dilution** | **Catalog number** | **Manufacturer** |
| --- | --- | --- | --- | --- | --- |
| miPEP133 | Rabbit | Western blot | 1:1000 | Custom | GenScript, Piscataway, NJ, USA |
| p53 | Mouse | Western blot | 1:1000 | 18032 | Cell Signaling Technology, Danvers, MA, USA |
| HSPA9 | Rabbit | Western blot  Co-IP | 1:2000  1:250 | A305-256A | Bethyl Laboratories, Montgomery, TX, USA |
| TOM20 | Rabbit | Western blot IF staining | 1:1000 (WB)  1:200 (IF) | 42406 | Cell Signaling Technology |
| TOP1 | Mouse | Western blot | 1:1000 | 556597 | BD Pharmingen, San Jose, CA, USA |
| Drp1 | Rabbit | Western blot | 1:1000 | 8570 | Cell Signaling Technology |
| p-Drp1 | Rabbit | Western blot | 1:1000 | 4494 | Cell Signaling Technology |
| MFN1 | Rabbit | Western blot | 1:1000 | 14739 | Cell Signaling Technology |
| OPA1 | Rabbit | Western blot | 1:1000 | 67589 | Cell Signaling Technology |
| TIM44 | Rabbit | Western blot | 1:2000 | ab201453 | abcam, Branford, CT, USA |
| VDAC1 | Mouse | Western blot | 1:1000 | ab14734 | abcam |
| HSP60 | Rabbit | Western blot | 1:1000 | Ab190828 | abcam |
| GAPDH | Mouse/HRP | Western blot | 1:10000 | HRP-60004 | Proteintech, Rosemont, IL, USA |
| β-actin | Mouse/HRP | Western blot | 1:10000 | HRP-60008 | Proteintech |
| Mouse IgG | Goat/HRP | Western blot | 1:10000 | SA00001-1 | Proteintech |
| Flag-tag | Rabbit | Western blot  Co-IP  IF staining | 1:1000  1:30  1:100 | Ab205606 | abcam |
| Ki67 | Rabbit | IF staining | 1:250 | ab16667 | abcam |
| Cleaved Caspase-3 | Rabbit | IF staining | 1:400 | 9664 | Cell Signaling Technology |
| Rabbit IgG | Goat/Alexa Fluor 594 | IF staining | 1:2000 | 8889 | Cell Signaling Technology |

**4) List of siRNAs:**

si-h-mi34-133_001: Sense, 5’ CUGGAAGCCUGCACUACCU dTdT 3’,

AntiSense, 3’ dTdT GACCUUCGGACGUGAUGGA 5’.

si-h-mi34-133_002: Sense, 5’ ACCUGGUCCUCUUUCCUUU 3’,

AntiSense, 3’ UGGACCAGGAGAAAGGAAA 5’.

si-h-mi34-133_003: Sense, 5’ UCUCGGUGACCACGCAGAU 3’,

AntiSense, 3’ AGAGCCACUGGUGCGUCUA 5’.

Control siRNA: Sense:5’UUUUCCGAACGUGUCACGUTT3’ ,

AntiSense, 5’ACGUGACACGUUCGGAGAATT3’.

5) **List of plasmid**

| **Name** | **Insert** | **Vector backbone** | **Application** | **Source** |
| --- | --- | --- | --- | --- |
| pcDNA3.1+ | None | pcDNA3.1+ | Transfection empty vector control  (Fig. 1b, 1d, 1e, 4a-f, and 5g) | Invitrogen (Carlsbad, CA, USA) |
| pcDNA3.1-miPEP133 | miPEP133 ORF (402bp) fused with flag-tag | pcDNA3.1+ | Overexpression of miPEP133 by transfection (Fig. 1b, 1d, 1e, 4a-f, and 5g) | Constructed in-house by cloning miPEP133 ORP from HEK293 cells into pcDNA3.1+ |
| PCDH-CMV-MCS-GFP-Puro | None | PCDH-CMV-MCS-GFP-Puro | Empty lentiviral vector control (Fig. 2e-q, 3a-i, 4g, 4h, 5a-j, and 6b-f) | System Biosciences (Palo Alto, CA, USA) |
| PCDH-CMV-MCS-GFP-Puro-miPEP133 | miPEP133 ORF (402bp) | PCDH-CMV-MCS-GFP-Puro | Lentiviral expression of miPEP133 (Fig. 2e-q, 3a-i, 4g, 4h, 5a-j, and 6b-f) | Constructed in-house by sub-cloning miPEP133 ORP from pcDNA3.1-miPEP133 into PCDH-CMV-MCS-GFP-Puro |
| pCMV-Neo-Bam | None | pCMV-Neo-Bam | Empty vector control (Fig. 5d) | A gift from Bert Vogelstein (Addgene plasmid # 16440 ; http://n2t.net/addgene:16440 ; RRID:Addgene_16440) |
| pCMV-Neo-Bam p53 wt | Wild-type p53 | pCMV-Neo-Bam | Overexpression of p53 (Fig. 5d) | A gift from Bert Vogelstein (Addgene plasmid # 16434 ; http://n2t.net/addgene:16434 ; RRID:Addgene_16434) |
| pCMV-Neo-Bam p53 R175H | p53-R175H | pCMV-Neo-Bam | Overexpression of mutant p53 (Fig. 5d) | A gift from Bert Vogelstein (Addgene plasmid # 16436 ; http://n2t.net/addgene:16436 ; RRID:Addgene_16436) |
| pCMV-Neo-Bam p53 R248W | p53-R248W | pCMV-Neo-Bam | Overexpression of mutant p53 (Fig. 5d) | A gift from Bert Vogelstein (Addgene plasmid # 16437 ; http://n2t.net/addgene:16437 ; RRID:Addgene_16437 |
| pCMV-Neo-Bam p53 R273H | p53-R273H | pCMV-Neo-Bam | Overexpression of mutant p53 (Fig. 5d) | A gift from Bert Vogelstein (Addgene plasmid # 16439 ; http://n2t.net/addgene:16439 ; RRID:Addgene_16439) |
| PG13-luc | Wild-type p53 binding sites and firefly luciferase | pBluescript II SK + luc | Evaluation of p53 transcriptional activity (Fig. 5j) | A gift from Bert Vogelstein (Addgene plasmid # 16442 ; http://n2t.net/addgene:16442 ; RRID:Addgene_16442) |
| pRL-TK | T7 promoter and Renilla luciferase | pRL-TK | Transfection efficiency control | Promega |
