# Supplementary material for: Identification of miPEP133 as a novel tumor-suppressor microprotein encoded by miR-34a pri-miRNA
Source: Mol Cancer. 2020 Sep 14;19:143. doi: 10.1186/s12943-020-01248-9 (PMC7489042; doi:10.1186/s12943-020-01248-9)
Supplement: Supplementary file 3 — Additional file 3. Description of data: This file includes Supplemental Figures S1-S5 [file 12943_2020_1248_MOESM3_ESM.pdf]

Supplemental Figures

S1

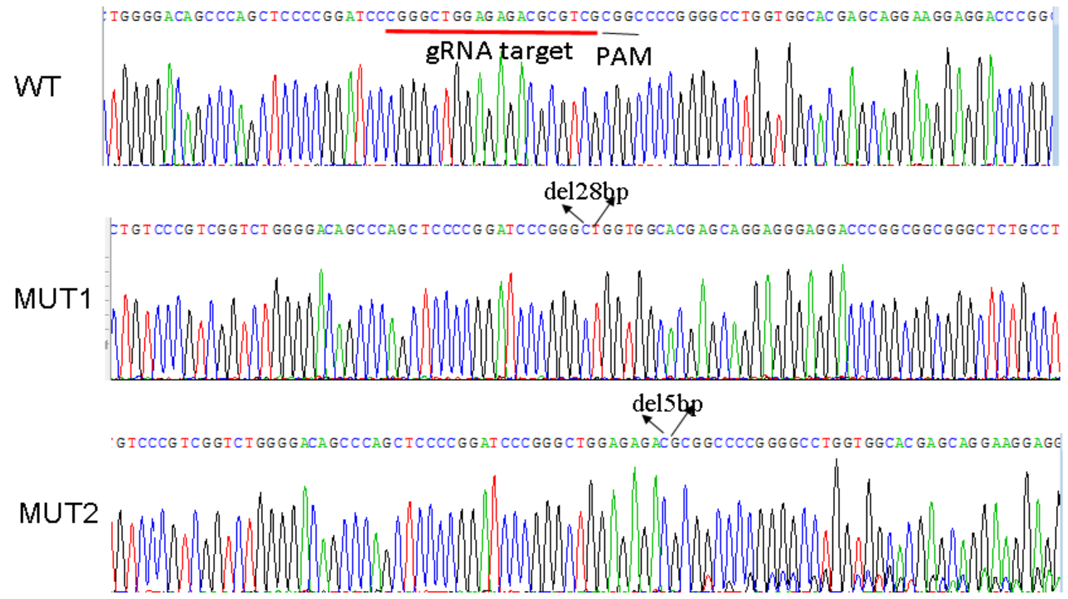

Protein

WT MPLSRRSGDSPAPRIPGWRDASRPRGLVA
RAGRRTRRRALPGLAWACSEPGCFSVTTQ
IGGIWRFCGSPAAKLRGRRGLEACTTCSP
RPSMHPGAGERQDRPVPRVPSGCRGPASC
ECFFGSVLAGCCEQ

MUT1 MPLSRRSGDSPAPRIP*

MUT2 MPLSRRSGDSPAPRIPGWR *

S2

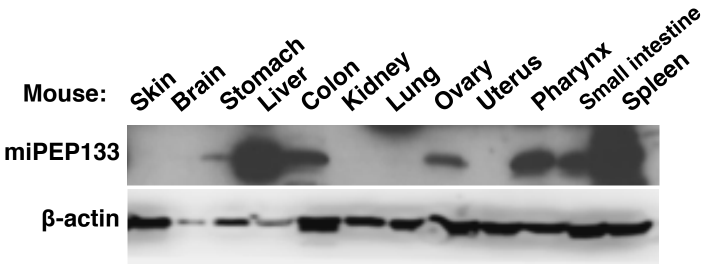

S3

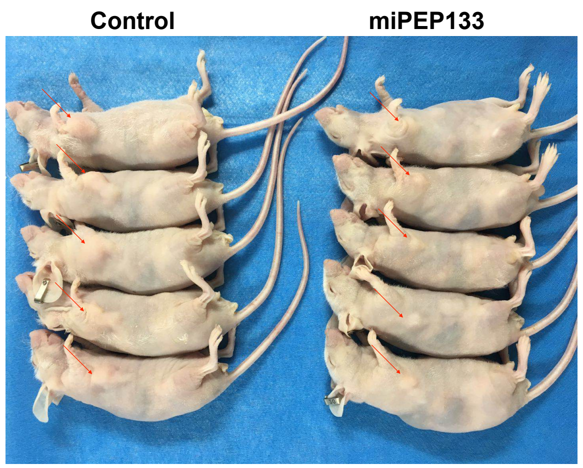

S4

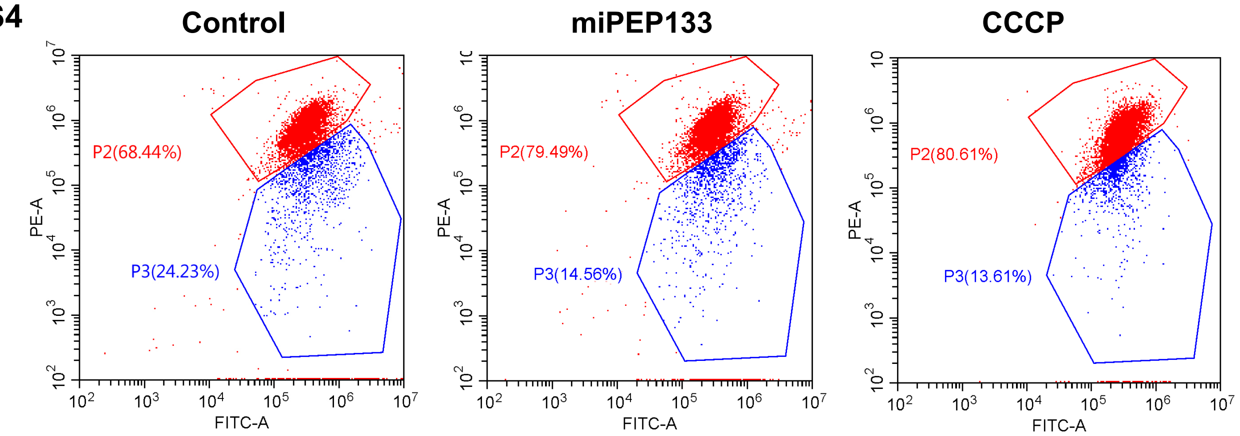

S5

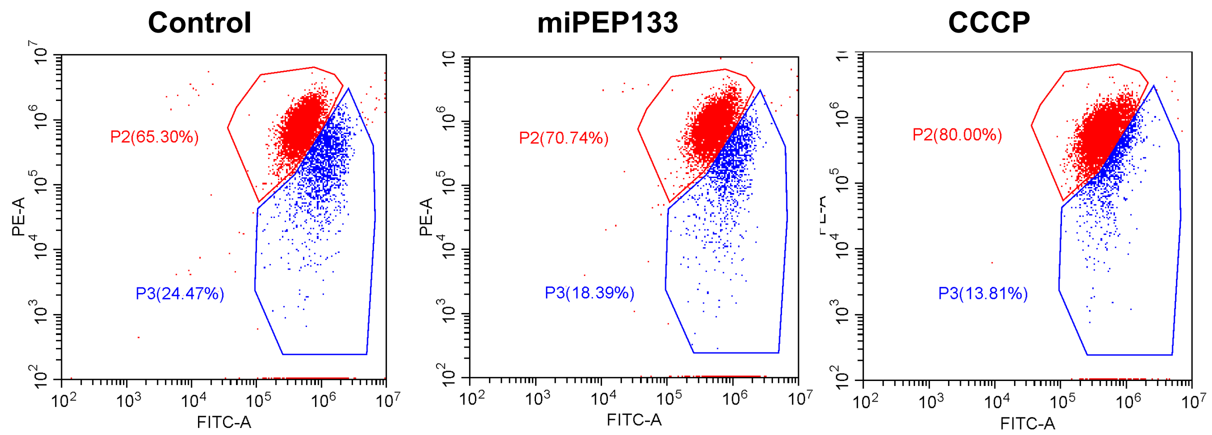

Figure S1. CRISPR/Cas9-mediated deletion of miPEP133 ORF. Sequencing results and the predicted sequences of the wild-type miPEP133 (WT) and the two mutant protein products (MUT1 and MUT2)

Figure S2. miPEP133 western blot of mouse tissue homogenates.

Figure S3. Nude mice with tumor xenografts formed by C666-1 cells. Control group was injected with C666-1 cells expressing control lentiviral vector. miPEP133 group was injected with C666-1 cells expressing lentiviral vector containing miPEP133.

Figure S4. JC-1 staining and flow cytometry analysis of HEK293 cells. Cells in control group were transfected with empty plasmid vector. Cells in miPEP133 group were transfected with miPEP133 plasmid. Cells in CCCP group were the control HEK293 cells treated with 50μM CCCP for 5 min.

Figure S5. JC-1 staining and flow cytometry analysis of Hela cells. Cells in control group were transfected with empty plasmid vector. Cells in miPEP133 group were transfected with miPEP133 plasmid. Cells in CCCP group were the control Hela cells treated with 50μM CCCP for 5 min.
